# 

**Published:** 1860-12

**Authors:** 


### BUSINESS NOTICES.

### GOODYEAR vs. TOLAND.

It is a source of much regret to me that the case of Goodyear *vs.* Toland, in the United States Court, for the Southern District of Ohio, in which I am charged with infringing the Goodyear Patent, by manufacturing or causing to be manufactured, Vulcanized Rubber Plates for Dental purposes, has not been decided upon the demurrer filed by myself in order to bring the case to a direct issue. Everything in my power has been done to push the matter, but the business before the court has been such as to preclude the possibility of reaching the case this term. I am extremely sorry that circumstances beyond my control have caused this delay. I wish my dental friends distinctly to understand that I am not dead, nor sleeping over their rights or my own, the case will be carried through by me, not from motives of malice or obstinacy, or from any hope of pecuniary benefits, that may accrue in event of a successful issue, for taking any view of the case, the result whether favorable or not—in that respect—will be a loss to myself, but actuated by motives of principle and duty, even though at a pecuniary sacrifice, I shall raise my voice against imposition, and protect as far as in me lies, the profession against such.

A great deal has been said about the American Hard Rubber Co. heretofore. I would not now introduce the subject again to the profession, but let the matter rest, until the termination of the contest with me—were it not that numerous letters have been received by me from my dental friends, asking advice as to how they shall answer threatening letters sent by the Agents of the American Hard Rubber Co., demanding payment for notes given for licenses, "whether they shall pay &c." these of course I can not advise, it is for them to determine how far the debt is due in point of conscience, and how far the fact by which it was promised, is or is not valid on account of the consideration. To those who have been threatened with suit for infringement, I can only hold out my course, leaving them to decide, without advice upon my part, whether to follow or not.

B. W. Franklin, the Agent of the American Hard Rubber Co., says in a letter to a friend, shown me, that "he" "the aforesaid Franklin, understands the matter—that he is satisfied beyond a doubt that Toland will be defeated." I mean no reflection on the gentleman by saying, that he may be mistaken—most men believe their own opinions to be correct. If a man were to go so far as to say, that he could conquer prejudice still it ought only to be admitted that he believes so—the frailty of human nature forbids complete confidence in any case. But here we have an exception—it was a great mistake upon the part of the American Hard Rubber Co., that they did not employ Dr. Franklin to conduct this case, and Dr. G. F. Foote to collect the testimony. I regret exceedingly for the sake of the Company, that Gall and Spurzheim, the eminent propogators of the philosophy called Phrenology are dead, for could they have surveyed the heads of this illustrious pair, they would declare "you are the men." I confess that in that case the result would have been doubtful, but since they have taken up with lesser lights, *I am* hopeful.

Whatever may be the result in this case, I feel sure, that this my determination and course, will never be regarded by the profession as a mark of arrogance to myself, but that they will on the contrary esteem it the strongest evidence that can be given of my fixed resolution, conscientiously to discharge my duty to them as well as myself. This determination was not taken without full knowledge of the difficulty and vexation to be encountered. I had too much knowledge and experience in human affairs not to foresee that it would bring me into conflict with the interests of a moneyed monopoly, which were perverting the rights of the profession and their common good to the advancement of its own particular prosperity. I have long since learned to hold in contempt all obstacles when in the discharge of what I believe to be my duty, and to hold my self as nothing and the rights of the profession everything.

The controversy between the American Hard Rubber Co. and myself in the outset, arose simply on the ground that they claimed more than their purported patent covered and though I have always been satisfied in my own mind, that their so-called patent was invalid and could be proved so if fairly contested, still I was willing to let the matter rest, if undisturbed myself, and would have done so, had I not been made a target of gross attacks, and subjected to repeated annoyance by their agents, which borne for a long time, patience at last ceased to be a virtue, and in fact directly charged with infringing, to use their own words, the Goodyear Patents, therefore, though willing at first to exhibit a spirit of forbearance, I am forced upon the defensive, either to admit the validity of the so-called Goodyear Patent, and totally disregard my honest opinions and convictions, or to take up arms and fight what I have always believed an imposition and sustain the voice of conscience and duty. What man believing this would or could have done otherwise? I am satisfied the law will not sustain the Goodyear claims. I am satisfied that the jury will not sustain them upon the facts unless, (I regret that the thought will force itself upon my mind,) the boasted wealth of the Company be used to pervert their honest opinions. I make no charge against the Company upon this ground, nor would a suspicion cross my mind, had not their agent boldly vaunted their untold wealth, and asked how I could expect to maintain my ground against this.

It has been my good fortune since the commencement of this controversy with the Hard Rubber Co., to meet with a number of persons well acquainted with the subject matter in dispute—men whose knowledge not only extends throughout this country but Europe, men whom I am confident will testify truly upon the witness stand. Such being the case, I flatter myself that my confidence in the success of this suit has not been misplaced. I speak advisedly.

That the controversy is one in which the Dental profession are interested, the letters I have received from various quarters are ample testimony; these letters are simply requests that I should make out subscription lists for funds necessary to defray the expenses of the contest, in which they offer liberally to subscribe. I am extremely thankful to the profession for their tangible regards and offers in this matter. The case, as I have said before, will be defended and carried to the utmost limit of the law by myself upon principles of duty and hatred of imposition. I shall neither seek or encourage any reimbursement for what I consider my duty and the rights of the profession; it is truly gratifying, however, to know that I am not alone in the love of justice and an opposition to all unjust *monopolies.*—" Let justice be done though the heavens fall."

TOLAND.

---

THE DENTISTS MEMORANDUM BOOK OF ENGAGEMENTS AND MANUAL OF READY REFERENCE, published by Jno. T. Toland, for 1861.

This is one of the most convenient little works ever published. Serving in the most convenient and economical way the appointments, as well as a record and account-book, and being a compendium of valuable formulas for references in daily practice.

Dentists, if they realize their own interest, will each of them, at once, send the price, ONE DOLLAR, and get one.

Don't read this unless you are deeply interested, otherwise you will find it a bore.

"VULCANITE—A. H. R. CO.—ROPES."

The November No. of the "Vulcanite" devotes ten pages of its valuable space to the re-publishing of copious extracts from all the various articles which I have from time to time written on the subject of vulcanite work and the Goodyear patents. This liberality on the part of the American Hard Rubber Co., deserves the thanks of the entire profession; as it places before them, in a compact form, the various stages of process, step by step, up to the present state of excellent simplicity.

The object is to attempt to show that I have at different times expressed different views on the subject—that I did not all the time keep and recommend the same kind of materials, &c.; but we should be willing to accept good from whatever source without questioning the object or intention.

It is true that I have changed, from time to time, as new light and greater experience developed new facts, or brought to my intelligence new ideas. *None but fools never change.* It is the part of wisdom to change by constant improvement.

There is not one word or sentence which I have written that I would now recall, but am perfectly willing to stand by the record; in so doing, I must not be held responsible for articles copied from other journals and written by other persons, nor for advertisements of other people's goods, written by themselves, without my endorsement. I kept the profession posted up, step by step, whilst others were "waiting for facts."

Two years ago, the Heater for vulcanizing was a cumbersome cast iron steam chest which could be used only in connection with a steam boiler; later, Warren & Banks' Heater was introduced and sold at $50, with innumerable joints, stop-cocks, &c., for Leaking, &c.; it was then thought ruinous to permit the flask to touch the water, next comes Roberts' Coal Machine, then the Gas and Alcohol Heaters; then it was found that it did no harm to let the flask get into the water; and more recently it was found, that to immerse the work entirely *under water*, not only saves time but makes better work; it seems now to be brought down to the most economical simplicity, and yet I will not be surprised if in another year improvements should occur which at present are not even thought of—nor should I then expect to be denounced because I had a year previous given all the information that could be had at that time, even though later a better and cheaper process might be discovered. In like manner with the Gums that have been introduced from time to time, I have placed them before the profession and stated what was *claimed* for them, I did not endorse the claims of one more than another, but have always explained the claims and my own views, that I have been misled, in one or two instances, for a brief period, I will not deny. Such was the case in August, 1858, when I stated that Mr. Goodyear's patent would soon expire and he was attempting to have it renewed, this statement was made on the authority of the *then agent* of the A. H. R. Co., whose letter is still on my file. I then went to work to investigate for myself, procured the specifications, and soon found my errors, and *as soon corrected* them. The same difficulty has occurred in regard to the Gums—a constant change and want of uniformity—changes of name as well as of characteristics—even the A. H. R. Co. scarcely succeed in producing two successive lots of Gum precisely alike; some are very fine, easily worked, and a bright color, whilst others are stiff, obstinate, dark and brittle.

The A. H. R. Co. give me *greater credit* than my own modesty would permit me to claim for myself, in stating that I have made my "pile," and that my business has more than quadrupled in the past year with large profits, &c., and that the *talents* employed by them is *confessedly inferior* to my own. Such eulogy I would scarcely expect from the warmest friend: in fact, the whole article is either directly or indirectly a recommendatory or eulogistic advertisement, the copious extracts from my articles give prominence and circulation to my views and opinions which could not have been obtained in any other way, and which I could not have had the assurance to ask; for all this I feel a deep sense of obligation which I feel I can never repay. With the exception of a few opprobrious epithets and invidious comparisons, (which only show their impartiality,) I doubt whether I could have written of myself so flattering a biography—and, as Dogberry remarks, "comparisons are odorous," I have no right to complain even of these—they are certainly making rapid strides toward having me to occupy that loving position toward the American Hard Rubber Co. which they so beautifully describe.

Mr. Ropes, *alias* the American Hard Rubber Co, is very anxious to impress upon the profession that my article, in the October No. of the Register, did not expose his "sophistry," but was "mainly devoted to attempts to cast doubts over the truth of our plain and unequivocal statement of facts." This matter I am perfectly willing to leave to the judgment of the profession, whose intelligent discrimination will doubtless be able to give a correct verdict without the necessity of forestalling their opinions, I would either consider my *arguments very weak*, or the *profession very stupid*, if I thought it necessary to follow up an article, by informing the profession how to decide between it and some one else's productions. My articles, once written, must take care of themselves; if they are good, the profession will adopt them; if bad, the sooner they are rejected and forgotten, the better. Does it look reasonable that, if my articles were as Mr. Ropes says, "mere groundless insinuations" and "garbling of extracts," they would have commanded the *influence* for which he gives me credit, it would ordinarily be supposed that an educated intelligent profession, embracing in its ranks some of the best minds in the country, would, during an intercourse of eleven years, have discovered, whether or not, my statements are worthy of confidence, without the aid of the interested representative of a wealthy monopoly whose only object in *suppressing me* is to *fill their own pockets* with the hard earnings of the profession in which they have no interest except to make the most money in the least time and at the least expense. Unfortunately for Mr. Ropes, he does not seem to understand that the Dental Profession attach more value to *plain truth* than to a beautifully worded article with well-rounded sentences; or, in other words, that they are peculiarly a class of men who

> "Seize upon truth where'er 'tis found,
> On christian or on heathen ground!"

I was not aware that the American Hard Rubber Co. had ever "made a statement of *facts*," or that they had *any facts* to state. True, they did publish an extract from the Goodyear patent specification, which claims, in substance, "the combination of India Rubber or allied Gums with Sulphur, either with or without the admixture of other ingredients in the proportions of from four ounces to a pound of Sulphur with a pound of Gum, to produce a hard and inflexible substance similar to Horn, Whalebone," &c. This is a CLAIM—*not a fact*—there is nothing immutable about this. True, a patent is in itself *presumptive* evidence of its validity until *legally contested* and *proved to be invalid*, but it is not a *fact*, until the highest tribunal in the country has pronounced it so, and even then the *claim may again be questioned* by another party, and upon additional testimony may be set aside—and when once set aside it is *invalid forever.*

Mr. Ropes is greatly exercised about my leaving off the latter part of the sentence in which he claimed that the "Goodyear patents cover all the vulcanizable compounds *which have yet been offered to the public for Dental purposes.*" There are no such words in the patent specifications. Yet in the same article, page 113, he says, "It is true, we did, in the May No. of the Vulcanite, in referring to the Goodyear patents, state that they covered *all vulcanizable compounds,*" but as we had in a preceding part of the same article in which this remark was made, published in full the claims of both of the Goodyear patents, *for the purpose of showing the extent of our rights,* it is absurd to suppose we intended anything more by our expression than was warranted by the patents themselves. But suppose we *did* intend something more than this, what harm could have resulted to the profession from such a claim. We had distinctly stated to them that *all the compounds then in the market for dental purposes,* including Amber Base, contained the proportions of ingredients described by Nelson Goodyear, and warned them against using them *for that reason.*" So the profession are to take the ipse dixit of the A. H. R. Co., and lay aside all free thought, investigation, or private judgment,

The A. H. R. Co. has declared that their patents cover all vulcanizable compounds—who dares dispute it—the whole course of this Company towards the Profession has been one of absolute dictation; in every article and every circular they convey the idea of themselves as "I am Sir Oracle, when I do ope my mouth let no dog bark!"

Here again, Mr. Ropes has mistaken the Profession—they require something more than broad assertions—these may do for the public with whom he has been in the habit of dealing, but a liberal Profession wants something upon which reason and judgment may take hold, so that each one can compare and determine for himself.

What claim has the A. H. R. Co. on the confidence of the Profession, that their single assertion should outweigh the better judgment of its own members against the evidence of the senses of every professional man. What interests have they in common with the Profession, except to make the most they can out of them as quickly as possible. "Marry in haste and repent at leisure," is a proverb that may well be applied to the purchase of patent rights.

Why, instead of stating these things in general terms, do they not give the precise constituents of the various *compounds?*

Let the Profession have the *formula* for giving the names and quantities of each ingredient in the American Hard Rubber Co.'s Gum.

I give the names as they are known in the market.

The same of Wheat's Compound,<br>

" " Amber Base,<br>

" " Enamel Gum,<br>

" " Metallic "<br>

Best India Rubber Composition.

Then we will have an opportunity to *compare notes* and talk to the point. Then will the Profession have an opportunity to compare and judge for themselves, without the aid of the superior judgment of the A. H. R. Co.

Mr. Ropes thinks it is of no practical importance to know who wrote the article in the August No. of the Vulcanite, nor whether Dr. Dieffenback deserted his wife, associated with women of easy virtue, and went to Europe with a young woman, leaving his family destitute, or not. It is a matter of common remark, that corporations have no souls, and this representative of the A. H. R. Co. seems to endorse the saying.

We think it of *great importance.*

1st. We wish to know the author of any article we read, that we may judge of his responsibility for truth, &c.

2nd. We like to know who we are talking to, that we may call persons by their *right names.*

3rd. When certificates are paraded before the Profession to influence their judgment for or against any article or doctrine, and especially when it involves a donation of $100 or more of cash or its equivalent, I think most men will agree that it is a matter of some practical importance to know who, or what manner of man it is, and I think it will also be conceded that, a man who will desert wife and family, leaving them destitute whilst he is revelling in voluptuous luxury with a mistress, is not the kind who should inspire the greatest confidence. Every man is presumed to be honest until he is proved dishonest.

When Dr. Dieffenback stated in his circular his claims for Amber Base, we believed him honest, and so treated him, but when he certified to his own misrepresentation, a doubt would naturally arise; and when further investigation proved what has previously been stated, we had no choice but to believe that "Birds of a feather flock together,"

"Mr. Toland asserts that Mr. Rob rts paid $100 only for *costs* and damages in the suit against him and received a receipt in full. This is either a deliberate falsehood, or Mr. Toland testifies to a matter of which he knows nothing"

"So in regard to Roberts continuing to vulcanize, we believe there is not the least foundation for the assertion, etc."

It is said that figures *do not lie.* Having seen and read the *receipt* myself, I can not easily doubt its correctness. It is in substance as follows:—

"Received, of E. A. L. Roberts, one hundred dollars in full, for costs and damages, in the case of Goodyear *vs.* Roberts, in the United States Court."

I am also credibly informed, that the actual Court costs in the case were over $75. So, therefore, they had for damages less than $25.

In regard to Roberts vulcanizing, it is simply a question of veracity between him and the A. H. R. Co., which they can determine to suit themselves.

There certainly could be no harm in the A. H. R. Co. purchasing heaters of Roberts, nor in supplying him gum for their licensees, but *my* remark was as to the *advertising* of Roberts's Heaters *immediately after* the settlement, which they *had not done before.* In justification they say "even Mr. Toland's name appears in our advertising columns every quarter." The difference is, that my advertisement is my own, and to be paid for at the regular price; but the one referred to is where the A. H. R. Co. put Roberts's goods into *their own advertisement.*

I make no pretensions, but have throughout stated plain facts. In all the first stages of the Vulcanite work, we knew so little about it, that the attention, waste of time, etc., caused more trouble and expense than was realized from the work, and from want of knowledge and experience, we, in common with others, made some very poor work; we were obliged to learn by investigation and experiment, as we could get no information from the A. H. R. Co., except that they had licenses for sale at certain prices, and on certain terms.

At first, we had considerable work to do, as every one wanted to try a piece, but as the profession gradually supplied themselves with the facilities to do their own work, the demand ceased. It requires nothing "artful" to dispose of such plain facts. There are very few if any, in the profession, in the West, who have had any work done, more than enough to satisfy them, whether or not they should purchase a Heater—the assertions of the A. H. R. Co. to the contrary notwithstanding.

I am now as ready and willing to do the work as ever, but for the reasons above named I do *not have it* to do. WHEN the United States Court has "whipped me in," I will certainly respect its decision, but I do not expect the United States Court, or any other tribunal, to require me to respect rights WHERE NONE EXIST.

The article in the August Number of the Dental Reporter referred to, was written under the influence of the best information then to be obtained, and with the exception of the statement that Goodyear was endeavoring to have the patent "renewed" instead of "re-issued," I have nothing to take back. He says—"We commenced the sale of office rights and apparatus on terms which it must be confessed were of a *liberal* character." This may or may not be so. Different persons view subjects from different stand points. The profession, from *their* point of view, can discover but little liberality in the matter.

The history of the suit with Christopher & Co., and Roberts, is indeed, familiar to the profession, and unfortunately reflects but little credit on either party engaged in the transaction. There is but little doubt in the minds of those who are supposed to be best informed on the subject, but that the "Coralite," under the patents of Rider & Murphy, would have been sustained as against the Goodyear patents, upon a fair trial. At that time I had not had an opportunity to investigate the patent claims, nor was there any occasion to do so. I believed then, and do still, that the Rider & Murphy patents stood upon the same platform with Goodyear's, so far as validity is concerned. Then I believed on the assumption of the parties interested, without investigation of my own, that they were all valid. Now, *after investigation*, I believe that neither of them can stand the test of law. This is the difference between then and now, or before (investigation) and after.

The "erroneous statement" in the July number, 1859, I have nothing to do with,—that portion of the *Register* is under the entire control of the *Editors.*

From the extracts taken from the December number, I quote only the italicised sentences, as follows:—"*This decision will probably bring the indiscriminate use of this process to a check.*" "*Those who wish to avoid difficulty should regard the rights of others,*" I now reiterate the above sentiments. The decision *did bring* the indiscriminate use of the process *to a check*, of which it was relieved by my notice in the January, March and June numbers of the *Register.*

I did not say that it *should* be checked, but merely prophesied that it *would.* And the prediction was verified; is there anything wrong in this? I am now ready to repeat the other sentence, viz: "Those who wish to avoid difficulty should regard the rights of others," and I might add, that it would, in many cases, be more economical to pay "black mail," or purchase a bogus right, than to take the chances of a law suit.

An old and experienced attorney once gave me, as a general advice, that if any one was indebted to me any amount not exceeding $100, and I could not collect without a law suit, it was best to give up the claim. And for this reason I have been particularly guarded in my expressions, so that the profession should not get into litigation through any advice or dictation of mine, and for that reason have refused t*o advise*, but of course gave what *facts* were in my possession, preferring to lose the sale of Heaters and materials, rather than have my friends get into difficulty. I have always displayed the darkest side, stating that they were liable to prosecution for the *first* piece of work made, and that it was at the *discretion* of the A. H. R. Co., to instigate suits to-morrow against every dentist in the United States, who is vulcanizing without license; whether or *not* they would *sustain a suit* is quite a different matter. I am clearly of the opinion that they can not. The next question is, whether it would not be cheaper to purchase a licence than get into a law suit, even if you should succeed; certainly it would, if dollars and cents is your guide, to the exclusion of professional position and principle.

For these reasons, I have purposely never expressed my opinions of the *weakness* of the Goodyear patents. as forcibly as it was impressed on my own convictions. I always prefer to err on the safe side, or if I do go astray, that none other shall be involved in my offence.

I have hundreds, yes, I think I might safely say thousands of letters of enquiry, desiring to know if I would guarantee, or warrant, or defend those purchasing machines or materials, from prosecution. My answer has always been an unequivocal—No, *Sir!* I give you the facts as far as I know of what has been done, and what is being done, the responsibility you must take yourself, as I have taken it, so far as my own course is concerned The majority have concluded to disregard the patent, and I can now say what I have not before stated, that I think they have drawn correct conclusions. Mr. Ropes labors hard to show that the course of the profession was shaped by *my influence.* This is too absurd to require an answer; the Dental Profession are not made of that kind of stuff to be influenced or modeled to the will of any individual. I furnished the facts, they formed their own resolutions.

The notice in the January issue is only a squib, confirming what before had been hinted at, and guarding the profession against the impositions which I then saw were about to be practiced on them under cover of the *compromise* decision of Goodyear *vs.* Roberts.

The introduction of Amber Base was first made through the New York *Dental Journal*, which at that time was owned and edited by an agent of the A. H. R. Co., and by circulars issued by Dr. Dieffenback. The first article in the *Register* was copied from the N. Y. *Dental Journal*, as a matter of general professional news. Subsequently, an arrangement was made between myself and Chas. Wehle, Esq., agent and attorney for Dr. Dieffenback, for the sale of Amber Base, and accordingly, an advertisement was put in the *Register*, made up of other advertisements, circulars, etc., with my name attached as Western Agent. The truth of the advertisement I am not responsible for, because it was made up from circulars, etc., which had before been before the profession, and the matter was endorsed by Mr. Wehle, who assumed to be an attorney as well as a patent agent, and he states to me by letter, that all the claims of Dr. Dieffenback were clear, and could be substantiated in court, and that they really desired an opportunity to contest the matter; under these circumstances, I could not refuse a general advertisement, and when in addition it was proposed to appoint me agent for the sale of materials, I could not well refuse to have my name attached to the *advertisement.* My selfishness in this matter consists simply in this fact, that knowing that the article was to be advertised, and anticipating that some of the profession would at least try it, I had *my own name* attached to the advertisement *as agent*, so that orders would come to me, or pass through my hands, leaving me a per centage instead of going to New York direct. With the *price* I had nothing to do; that $14 per pound was an exhorbitant price I will not deny, but I did not fix the price, but received a per centage on what I sold, as I do on the A. H. R. Co's gum, and all *others*, and the aggregate profits on the Amber Base were less than that of any other vulcanite material which I have had for sale.

Mr. Ropes says "We charge also that since January, 1860, Mr. Toland has not repeated, in his journal, *one word* of the caution which he uttered as to the infringements on patents previous to that date.

Why should I? Having once placed the matter fairly before the profession, I did not deem it necessary to reiterate it in each successive number, as if I were teaching a class of children, or as if, like Mr. Ropes, I had but *one idea.*

The extracts from the March number of the *Register* are well chosen, and convey all the information we had at the time. Mr. Ropes says "can any one fail to see that the former part of that article was written for the purpose of strengthening the positions taken in the latter part, and that under the guise of an apparent regard for patent rights, Mr. Toland was only endeavoring to change the course of trade from an exploded material to a new one, in which he had a large interest.'' Could sophistry be more complete. The whole article was written together, giving the profession a fair and impartial review of *all sides*; and whilst I have nothing to say now in defense of Dieffenback and his confederates, he had the same right to be heard that any other had. If he misrepresented the claims of his patents, he only followed in the footsteps of illustrious predecessors. My endeavour to change the course of trade amounts only to the fact that, as soon as I discovered any article to be worthless, or overrated, I not only ceased to recommend it, but at once denounced it.

In regard to the *plaster moulds*, I quoted from Dieffenback on trust, because I considered it a matter of very small consequence whether or not the plaster mould patent existed. If all the other paraphanalia of these patents are of no force, the use of plaster paris will scarcely interfere with a profession with whom plaster paris is a *text*.

That Dieffenback backed down from his position I am free to admit, and can further state, that I was the first to *denounce* him in consequence; but we are not limited to either the claims or testimony of Dr. Dieffenback, for the defeat of the plaster mould patent, or the vulcanite.

I do not claim omniscience; I have been sometimes mistaken when I was dependant on others for information, whose honesty I could not then doubt, but the profession thus far has never suffered from these little errors, because they have always been *promptly corrected*, as soon as I had any satisfactory evidence of change of facts or circumstances. Mr. Ropes thinks it a "severe blow" to me to find that Dieffenback had misrepresented his claims to the profession, and that at the first approach of danger he backed down and "sold out." But what else could be expected from such a character, the only misfortune was that we did not learn the *character* of the man until after he had done the mischief, in this we labored under the same difficulties as do the public, who have their pockets picked, stores robbed, or themselves swindled, by placing too much confidence in those supposed to be friends. Mr. Ropes seems to regard permanency of ideas as everything, and honesty as nothing. The entire ten pages is devoted to showing that I did not express precisely the same views in each successive number of the *Register.* He can not apparently appreciate that kind of honesty and independence which enables a man to acknowledge a fault, and correct an error, as publicly and promptly as he would make a declaration of conscientious convictions.

"Can't you see, sir, that what you have put forth as an argument against the validity of the Goodyear Patents is one of the strongest which can be made in their favor?" No, sir; not at all. The patents to John Rider and John Murphy were issued subsequent to the original patent of Nelson Goodyear, (which was by Henry B. Goodyear acknowledged to be *inoperative*) and prior to the RE-ISSUE. Why then did not the commissioner refuse to grant patents to John Rider and John Murphy? or why, having granted these, did he grant a re-issue to Goodyear? The A. H. R. Co. claim that the patents of John Rider and John Murphy are infringements on the Goodyear Patent.

It is news to me to learn that I have made a great "*hue and cry*" about their claiming more than their patents covered. I stated such to be the fact, and now *re-iterate it*, and also repeat that therein was the point of controversy, until they, in their wisdom, resolved to stop my voice and pen by bringing suit against me.

Now there are two *issues*, one is the controversy, as to their claiming more than their patents cover—the other is the *direct* and *positive denial of the validity* of the patents of Nelson Goodyear. I am free to declare, that in my judgement and opinion it is *void* of the chief requisites of a patent, that it possesses neither *originality* nor *novelty*, and has not been in use long enough to entitle it to a claim to *utility*. When the United States Court has succeeded in "whipping me in," I may change my views, until then they will remain as they are.

The first point *has been fully discussed;* the latter I will leave to my attorney.

One of the great mistakes of the A. H. R. Co. has been their arbitrary mode of doing business, requiring the profession to buy "a pig in a poke," on their simple *assertion*, without any testimony, circumstantial or otherwise, to corroborate. They say—"We had distinctly stated to them, that all the compounds then in market for Dental purposes, including Amber Base, contained the proportions of ingredients described by Nelson Goodyear, and warned them against using them for that reason." But did the A. H. R. Co. place before the profession any reasons why their simple assertion should be believed, and that of other interested parties should be disbelieved. Did they at any time state the composition of these various compounds, either from chemical analysis, or from any other source of knowledge.

Mr. Ropes is very anxious that I should, in a public manner, expose all the facts and circumstances of knowledge, in my possession, affecting the validity of the Goodyear Patents, to enable him to fortify himself before the day of trial. Before suit was entered, it would not have required much urging to prompt me to give quotations of publications and oral testimony, which, I think, would convince any impartial mind of the invalidity of the Goodyear Patents.

I have, and do still propose to vulcanize for the profession. The A. H. R. Co's gum being the lowest priced article in market, it is but fair I should vulcanize it for less than gums which cost 2, 3 or 4 times as much.

That my business has greatly increased I will not deny, but that the increase is any way dependant on the vulcanite work, more than on any other element of business, is too absurd to require notice, and I have yet to learn that to be successful in business is to be censured.

The A. H. R. Co. had confidently expected that their COMPROMISE with Roberts and *Dieffenback* would remove every obstruction between them and the *pockets* of the profession, and are sorely disappointed to find that the profession could not be *gulled* by so transparent a *trick*, and must, of course, vent their spleen on some one else; they assume, therefore, that I have made all that they have lost.

"Now, a few words to those who have yielded to his influence." Here comes in the solemn warning, with a new edition of the *old threats*. They are at last about to dispose of that fund of $150,000 (which has so long been lying idle) by immediately entering suits against one thousand or two thousand Dentists, in different parts of the country. They are about to perform a miracle by extracting blood from a turnip. The time will soon arrive when they are resolved to have their "*pound of flesh.*" But let them *beware* that they spill not one drop of BLOOD. They say—"We have found DENTISTS generally *too* POOR to squander money in law suits, and have advised them, therefore, to pay their $100, where it will secure them from all liability to such suits." That is, in other words, "Your *money* or your *life*." The A. H. R. Co. here again exhibit their ignorance of the profession; they will find them, as Tom Marshall remarked in the Ward trial at Louisville, in regard to the people of Kentucky, they are "poor and proud, and the poorer the prouder."

"A few words to Mr. Toland, and we have done. You have, without just cause, used your GREAT INFLUENCE, through your *widely circulated Journal*, to destroy our right and deprive us of our property. In defending those rights, and endeavoring to protect that property, we have employed TALENT, *which, though confessedly inferior to your own*, in certain directions we thought would answer for the occasion." * * * *

*Prodigious!!* I never would have known what a great man I am but for the kindness of the A. H. R. Co.

It is but a few months ago, that myself and the profession were informed, through the N. Y. Dental Journal, by the agent of this Co., that the Register was losing the respect of its subscribers and the profession, also, that the respectable portion of the profession would not sympathize with me in my opposition to Dental patents, they certainly have a queer way of showing their facts.

Now they give the Register the credit of a *wide circulation*, and represent that my business has more than quadrupled in one year. This is a new way for the profession to exhibit their disrespect and want of sympathy: they do not offer their agents and attorneys much of a compliment by confessing their inferiority.

I have no doubt that the A. H. R. Co. would take some satisfaction in seeing me at a rope's end, quartered as well as hung. I was not aware that doubting the validity of a patent cautioning the profession against imposition, or refusing to recognize claims which I am convinced have no real foundation, was a hanging offense; but "I was born to be drowned." Our acquaintance however has been too pleasant to be terminated until I and the profession have the satisfaction of seeing them at the end of their Ropes.

Mr. Ropes censures me for the large amount of business done in the sale of Vulcanizers and Gums, and yet in all their articles and advertisements they acknowledge that any one has a right to manufacture and sell these.

How this can be construed against me I do not understand. The Vulcanizers of my own make were sold lower than the same kind could be purchased any other place, whilst the Gums were also sold at the same price as elsewhere, as I never manufactured a pound of the gum in my life, but simply bought and sold, making a per centum on the sale the same as on other goods

The facts are just the reverse. Dentists, like other people, purchase their goods where they CAN DO BEST; it was because they got *better goods for less money*, with more information and instructions than they could elsewhere, which induced the profession to purchase of me in preference to others.

The low price of the A. H. R. Co.'s Gum is simply a bait held out as a temptation to purchase a right, why, if their rights are so perfect, did they enter into competition on prices of vulcanizing, and as I am informed, reduced the price to 50 cents a piece until others abandoned it, then again resumed the old price.

An array of certificates is published in the Vulcanite, in regular quack medicine form, but I doubt if even this dodge will win as I can hardly believe the profession could so soon have forgotten how they were taken in with "cheoplasty"—yet they object to have their *secret compounds* called a NOSTRUM!

I regret the necessity of taking up the space of the Register, or taxing the time of the profession, and trust this will be the last article of any length on this subject. I think the whole ground has b^en covered, and therefore needs no further discussion except that which our attorneys will have to do before a jury of our peers in the United States Court—the result of which has never caused me a doubt.

I have never asked the profession to adopt my views or take sides with me, I have taken my ground without advice, and will maintain it on my own responsibility; but none can fail to see that in a controversy and contest of this kind "*those who are not* FOR ME ARE AGAINST ME."

I am a little in doubt how to close this article, but as it seems according to the A. H. R. Co., an *offense* to do a LARGE BUSINESS, and as I have devoted myself to tbe interests of the profession, probably it would be best to advise you all to purchase your goods elsewhere, where you can have an opportunity to PAY MORE for them,—as it seems to be a hanging offense to quadruple one's business in one year, to double it again in the next, must be a crime for which the laws provide no adequate punishment.

I am as ever, yours truly, JOHN T. TOLAND.

In conclusion, The A. H. R. Co will find me something like a certain witness who in a magistrate's court had stated some facts not very palatable to the magistrate, who in the cross-examination asked the solemn question, Are you fully aware of the solemnity and obligations of *an oath!!* Witness leaned back, looking the magistrate full in the face, exclaimed, Look here, Squire, "I DON'T SCARE WORTH A DARN!!"

NOTE.—The above article was written after the article headed Goodyear *vs.* Toland had gone to press,—hence the delay. I hope in succeeding Nos. of the Register to publish such FACTS as will LEAVE NO DOUBT in the minds of the profession.

---

PARAFFINE.—By late numbers of Eastern publications, we find that this article is just being introduced in the East. We, in the West, have given it a fair trial, and abandon it as worthless—for the purposes designed.

THE LAMP has again shed its lurid glare over the professional world. It has many good things which recommend it to the profession. We have but one objection to it, being the constant attempt to prostitute the business, by advertising low prices, which can be sustained only by the sacrifice of quality.

REDUCTION OF PRICES.—Our neighbor, Dr. Brown, calls attention to *reduction of prices* and his claims for patronage, whilst I will not say one word in disrespect of the gentleman. It is *simply absurd* to think or talk of his ability to purchase or sell goods lower than others. The laws of trade gives to the heaviest dealers the advantage of price and time. My sales of Teeth and other goods, are from THREE TO FOUR times as large as those of any other house west of the mountains, and I think no one can doubt my ability to purchase and sell at LEAST AS LOW as any other house. Indeed my business has grown to its present *magnitude*, simply because the profession found that their *interests were better served* by purchasing of me than elsewhere.

OUR MEMPHIS DENTAL DEPOT has long since ceased to be an experiment. It is now an established institution with the flattering prospect of soon being second to none in the United States in its resources and patronage.

Under the management of Dr. S. C. Gray, whose personal interests and attention, with ripe experience, are devoted to the interests of the profession through a well regulated Dental Depot, and who being a Southern man, and in every respect familiar with the wants of the profession in the South, the Memphis Dental Depot cannot fail in blending the interests of the profession with its own in mutual advantage.

**Prepared Cotton for Dental purposes,**—A good article, and reliable absorbent for drying cavities, price 20c and 25c.

**Corundum Tape,**—A superior article which saves much time in finishing filling,. price, 10 cents.

**Ask's Patent Dental Chair,**—At the manufacturer's prices.

**Spunk for drying cavities,**—Valuable in absorbing moisture in sensitive teeth, as the patient does not experience the pain produced by the application of paper or cotton, price, 20 cts.

**New Electrio Galvanic Battery,**—A small, neat battery, alike useful in extracting teeth and electroplating, price, $10.

**Portable Forge and Bellows,**—For Dentists and Jewellers, price, $22 and $27.

**Anatomical Preparations,**—First and second Dentition, $12, ——— Exhibiting Arteries, Veins and Nerves, $20.

**Richardson's Mechanical Dentistry,**—Just out, with 100 illustrations, price, $3 25.

**Taft's Operative Dentistry,**—The only practical treatise published, well illustrated, price, $2 75.

**New Pure Amalgam,**—Prepared at head quarters, and put up in ONE DOLLAR, packages with full directions to insure the best results.

**Teeth,**—Just received from JONES & WHITE the finest assortment of Teeth ever opened in the West for sale.

**Vulcanite Heaters,**—On hand and for sale the best and cheapest vulcanizer in the country. See advertisements.

**American Hard Rubber Co.'s Gum,**—For Licensees only, all other

**Vulcanite Compounds,**—Sold without restriction.

**Osteoplastic,**—Or Artificial Bone, $1, $3, and $5.

**Roberts' Os-Artificial,**—A compound mineral filling in great demand, price, $1.

**Artificial Dentine,**—A compound of Gutta Percha similar to Hill's stopping for temporary fillings, price, $2 per box.

**Moulding Flasks,**—Hawes, Luthers and all others.

**Dentist's Chairs,**—Every variety of style and pattern, from $25 to $125.

**Spittoons,**—Of Rosewood and Mahogany from $12 to $160.

**Lathes,**—With stand for grinding and polishing, Hand Lathes, Foot Lathes and Bench Lathes, at all prices.

**Travelling Cases,**—Of all kinds and descriptions, and at all prices.

**Instrument Desks, or Office Cases,**—A new and beautiful article varying in price from $65, to $150.

**Dental Register of the West,**—Published monthly at $3, a year, STRICTLY IN ADVANCE; the volume commences with the year, those desiring it sent to their address for the coming year will please send in their address with the subscription price without delay.

BOUND COPIES OF THE DENTAL REGISTER OF THE WEST, Vols. 13, 14.

### FRENCH INSTRUMENTS.

### EXCAVATORS, DRILLS AND PLUGS.

I have now on hand and have arranged to receive regular supplies, direct from the manufacturer, a full assortment of these excellent instruments.

After years of annoyance with inferior excavators and drills, which would either break the first time they were used, or be so soft as to be always dull, and plugging with imperfect points badly surrated and worse tempered, the profession can now appreciate instruments in this line Excavators with an edge like a razor burs, cut with perfect regularity, and pluggers surrated with clean, clear, distinct, sharp points, every point of equal length, and yet so delicate as scarcely to be visible to the naked eye, and withal the *temper perfect*, so as neither to break or bend. The prices are something above the ordinary price, but would be cheap if twice as high as they are

| | | |
|---|---|---|
| Excavators and burs | $2 00 per dozen. | |
| Fang Excavators and pluggers | 2 50 " | |
| Small Octagon Wire-Headed Pluggers | | $3 00 |
| Octagon File Cut Pluggers | | 5 00 |
| " Ivory " | | 10 00 |
| Steel Handled Chisels | | 4 00 |
| Large Steel Handled Burs, for dressing fillings | | 7 50 |

***Jno. T. Toland.***

### BALTIMORE COLLEGE OF DENTAL SURGERY.

**TWENTY-FIRST ANNUAL SESSION 1860-61.**

### FACULTY.

CHAPIN A. HARRIS, D. D. S., M. D.
*Principles of Dental Science and Practice of Dental Surgery.*

THOMAS E. BOND, M. D.
*Therapeutics and Materia Medica.*

PHILIP H. AUSTEN, D. D. S., M. D
*Mechanical Dentistry.*

REGINALD N. WRIGHT, M. D.
*Chemistry and Metallurgy.*

A. SNOWDEN PIGGOT, M. D.
*Anatomy and Physiology.*

CHRISTOPHER JOHNSTON, M. D.
*Microscopical and Comparative Anatomy of the teeth.*

FERDINAND J. S. GORGAS, D. D. S.,
*Demonstrator of Mechanical Dentistry.*

SAMUEL T. CHURCH, M. D., D. D. S.
*Demonstrator of Operative Dentistry.*

The twenty-first Annual Session will commence on the 1st. day of October, and close on the 1st of March.

The regular course of LECTURES will commence on the 1st of November

The entire day during October, and the afternoon of each day during the rest of the session, will be devoted to Practical Dentistry and Anatomical Dissections.

The Infirmary has a large and rapidly increasing supply of patients, affording to the student unsurpassed facilities for the acquirement of a PRACTICAL knowledge of every Operation in Surgical Medical and Mechanical Dentistry.

Professors' Tickets, $100 : Demonstrators' Tickets, $20 ; Matriculation Fee, $5 ; Diploma Fee, $30. Dissection Ticket, (optional), $10.

For further information, address **P. H. AUSTEN**, DEAN OF THE FACULTY,
79 North Charles Street, Baltimore.

### PENNSYLVANIA COLLEGE OF DENTAL SURGERY

SESSION 1860-'61.

### FACULTY.

T. L. BUCKINGHAM, D. D. S.,
Prof. of Chemistry and Metallurgy.

J. H. McQUILLEN, D. D. S.,
Prof. of Anatomy and Physiology.

WILLIAM CALVERT, D. D. S.,
Prof. of Mechanical Dentistry.

C. N. PIERCE, D. D. S.,
Prof. of Dental Physiology and Operative Dentistry.

J. L. SUESSEROTT, D. D. S.,
Prof. of the Principles of Dental Surgery and Therapeutics.

D. H. GOODWILLIE, D. D. S.,
Demonstrator of Operative Dentistry.

J. J. GRIFFITH, D. D. S.,
Demonstrator of Mechanical Dentistry.

The regular Course will commence on the first Monday of November, and continue until the first of March ensuing.

During October, the Laboratory will be open, and a Clinical Lecture delivered every Saturday by one of the Professors, at 3 o'clock P. M.

The most ample facilities are furnished for a thorough course of practical instruction.

Tickets for the Course, Demonstrators' Tickets included, $100; Matriculation Fee $5 Diploma Fee $30. For further information, address W. CALVERT, DEAN,
133 North Eleventh Street, PHILADELPHIA.

